# Supine sleep position and angina frequency: an analysis from the sleep heart health study

**DOI:** 10.1007/s11325-025-03501-1

**Published:** 2025-10-22

**Authors:** Jeongok G. Logan, Ha Do Byon, Yeajoo Lee, Yeonsu Kim, Sunbok Park, Younghoon Kwon

**Affiliations:** 1 University of Virginia School of Nursing, 202 Jeanette Lancaster Way, P.O. Box 800782, Charlottesville, VA 22903 USA; 2 University of Virginia Health System, 1215 Lee Street, P.O. Box 800782, Charlottesville, VA 22903 USA; 3 College of Nursing, The Catholic University of Korea, 222 Banpo-daero, Seocho-gu, Seoul, 06591 Republic of Korea; 4 Division of Cardiology, University of Washington, Harborview Medical Center, 325 9th Ave Main Hospital, WA 98104 Seattle, USA

**Keywords:** Habitual sleep position, Angina pectoris, Chest pain, And cardiovascular risk

## Abstract

**Purpose:**

Habitual sleep positions might influence cardiovascular function, but its association with cardiovascular risk in the general population remains unknown. This study aimed to examine the relationship between the supine sleep position and the frequency of angina pectoris (angina), a common symptom of ischemic heart disease, and to explore whether body mass index (BMI) or sex moderate this relationship.

**Methods:**

Data from the Sleep Heart Health Study (SHHS) were used. Supine sleep position was quantified as the percentage of total sleep duration spent in the supine position, measured by unattended home polysomnography (PSG) during the first SHHS visit. Angina frequency was defined by the number of episodes over a 15-year follow-up period. Multiple Poisson regression was conducted, adjusting for various conventional cardiovascular disease (CVD) risk factors.

**Results:**

The study included 5,804 participants (52% female), with a mean age of 63 years and an average BMI of 28.16 kg/m^2^. The average time spent in the supine position was 32.4%, and the mean number of angina episodes was 0.48. Longer time in the supine sleep position was independently associated with an increased risk of angina (IRR = 1.003, *p* < .0001); a 10% increase in supine sleep time was associated with a 3% increase in angina risk. BMI (*p* = .189) and sex (*p* = .117) did not significantly moderate this association.

**Conclusion:**

Sleeping in the supine position was significantly associated with more angina episodes. The study lacked information on other sleep positions. Identifying optimal sleep positions to support cardiovascular health could significantly contribute to CVD prevention.

## Introduction

 Angina pectoris (angina) is the most common symptom of ischemic heart disease, primarily caused by coronary artery disease (CAD) [1]. Angina, characterized by chest pain and discomfort, occurs due to an imbalance between the oxygen supply to the myocardium and its demand [1]. Angina is classified into stable and unstable types. In stable angina, increased oxygen demand leads to insufficient blood flow to the heart muscle during physical exertion or emotional stress. In unstable angina, these episodes can occur even at rest [2]. In the United States (U.S.), approximately 10.8 million people suffer from angina, with prevalence increasing with age in both men and women [3]. The burden of angina extends beyond physical discomfort, leading to emotional distress, limitations in daily activities, and a heightened risk of adverse cardiovascular events such as myocardial infarction, stroke, and heart failure [4]. Thus, angina represents a significant public health challenge, requiring comprehensive prevention strategies to mitigate its far-reaching consequences and improve the well-being of affected individuals [5–7].

The frequency of angina episodes has been associated with various demographic and clinical factors, such as age, psychological stress, depressive symptoms, body mass index (BMI), a history of smoking, physical inactivity, dyslipidemia, hypertension, and diabetes [1, 2, 8, 9]. Previous studies have highlighted the significant impact of sleep features – such as sleep quality, sleep duration, and sleep architecture - on cardiovascular disease (CVD) risk [10–15]. In particular, population-based studies have reported that individuals with either short or very long sleep duration, as well as poor sleep quality had a higher risk of stroke and major coronary events [10, 16, 17]. Given the critical role of sleep in CVD risk, it is essential to consider all sleep-related factors that might influence this risk. Although research on the effects of different sleeping positions on cardiovascular function has produced mixed results, most findings suggest that the supine position is associated with increased sympathetic activity, higher stroke volume, and greater cardiac output compared to other positions [18–20]. These studies may suggest that specific sleep positions could potentially elevate the risk of CVD such as angina. However, the link between habitual sleep positions and angina is largely unexplored.

This study aimed to explore the association between the supine sleep position and the frequency of angina episodes. Considering the potential influence of BMI on chest pressure during the supine position and the well-known sex difference in angina prevalence [21, 22], we also investigated whether BMI and sex moderate the relationship between supine position and angina. We hypothesized that individuals spending more time in the supine position during sleep may experience a higher number of angina episodes, and that BMI may moderate this relationship. By clarifying the potential link between supine sleeping and angina episodes, our study sought to enhance our understanding of a potential modifiable risk factor for angina, thus informing clinical practice and improving patient care strategies.

## Methods

### Study population

The data for this study were obtained from the Sleep Heart Health Study (SHHS) database (https://sleepdata.org/datasets/shhs) [23]. The SHHS, a multi-center prospective cohort study, was designed to investigate the cardiovascular outcomes of sleep-disordered breathing. The participants were recruited from nine existing cohort studies and the SHHS incorporated the assessment of sleep and sleep-disordered breathing into their protocols. Prior publications have outlined the study’s design. A baseline examination conducted between 1995 and 1998 involved 6,441 men and women aged 40 years or older, However, 637 participants withdrew their consent due to sovereignty concerns, resulting in a dataset comprising 5,804 participants. Cardiovascular disease outcomes data were monitored and evaluated by the parent cohorts from baseline until 2011. Institutional Review Board approval was secured from all participating institutions, and informed consent was obtained from all participants at the time of their enrollment into each stage of the study [23].

### Sleep assessment

In-home sleep studies using one night of unattended polysomnography (PSG) (Safiro, Compumedics, Inc., Melbourne, Australia) were administered by trained technicians. Home visits were conducted by mixed-sex teams and lasted between 1.5 and 2 hours. Special attention was given to ensuring that the night of polysomnographic assessment resembled a typical night of sleep for the participant. Participants were instructed to schedule the visit approximately 2 hours before their usual bedtime, and efforts were made to maintain consistency in participants’ bedtime routines, whether it was a weekday or weekend, corresponding to the day of the week the visits occurred.

All sleep parameters were obtained from type II PSG during the baseline examination of the SHHS. The SHHS recording montage included electroencephalogram, electrooculogram, submental electromyogram, plethysmography for thoracic and abdominal movements, airflow (measured by a nasal-oral thermocouple), pulse oximetry, electrocardiogram, body position (via a mercury sensor), and ambient light (monitored by a light sensor). A certified technician placed the sensors and calibrated the equipment during a home visit. After data collection, the information was stored on Personal Computer Memory Card International Association cards, downloaded to local computers, reviewed, and sent to a central reading center (Case Western Reserve University). Sleep scoring followed Rechtschaffen and Kales guidelines, ensuring consistency across centers, with high reliability between scorers [24, 25].

*Supine sleep position* was determined by calculating the percentage of total sleep time spent in the supine position. *Sleep Efficiency* was assessed by dividing total sleep time by the total time spent in bed, expressed as a percentage. *Total Sleep Time* (TST) denotes the duration between falling asleep and waking up, while time in bed represents the duration between getting into bed and getting out of bed. *Sleep apnea* was evaluated using the apnea-hypopnea index (AHI), which is calculated as the total number of apneas characterized by at least a 3% decrease in oxygen saturation with or without arousal, along with hypopneas characterized by a more than 30% reduction in airflow and at least a 3% decrease in oxygen saturation with or without arousal, per hour of sleep.

### Outcomes

The frequency of angina was defined as the number of angina episodes occurring from baseline PSG to the 15-year SHHS follow-up period. CVD outcomes including the number of angina episodes were monitored and adjudicated by parent cohorts between baseline and 2011. Each study supplemented its standard surveillance practices by sending mailings to all SHHS participants who had not been contacted by the parent study within 12 months of the final follow-up date. The diagnosis of angina was based coronary ischemia syndrome, ECG findings, results from exercise stress test, and coronary angiography findings, obtained during the follow-up CVD outcomes assessments [26].

### Sociodemographic and clinical data

Other related information, incorporated to this study as covariates, included sociodemographic characteristics (age, sex, and race), lifestyle habits (smoking and psychological stress), clinical conditions (BMI, systolic blood pressure (SBP), and cholesterol), comorbidities (history of diabetes, coronary artery bypass grafting [CABG], and coronary angioplasty), and medications (use of oral hypoglycemic medications, use of insulins, anti-hypertensive medications, lipid lowering meds). Information on sociodemographic characteristics, lifestyle habits, comorbidities, and medications were obtained through interviews. Smoking status was classified as “never” (if the participant reported lifetime smoking of fewer than 20 packs of cigarettes), former, or current. Stress was classified as “a typical day”, “less stressful than usual”, “more stressful than usual” to the question, “how stressful was day today?” Weight and height were measured by a portable scale after the subjects had fasted overnight and were wearing only underwear during the home visits. BMI was calculated as weight (kg)/height(m^2^). Blood pressure (BP) was measured according to a standardized protocol with a standard mercury sphygmomanometer on the left arm after 10 min of rest. The information for serum cholesterol levels was obtained from the existing parent studies. The SHHS manual of operations contains detailed information regarding the protocols for these measurements [23].

### Statistical analysis

For descriptive statistics, means and standard deviations were computed for continuous variables, while frequencies and percentages were calculated for categorical variables. For inferential statistics, simple Poisson regression was used to examine bivariate associations, and multiple Poisson regression was conducted to examine associations involving more than two variables and explore the moderation effect. Moderation was tested including interaction terms in regression models. Poisson regression analysis was selected because the outcome variable, frequencies of angina, is a count variable that is right-skewed. The outcome variable was not over dispersed. For Poisson regression, incidence rate ratios (IRR) and the 95% confidence intervals (CIs) were computed by exponentiating the regression coefficients. All data analyses were conducted using SPSS version 29 (IBM Corp, Armonk, NY) and statistical significance (alpha) was set 0.05.

## Results

The analytic dataset included data from 5,804 participants. Table 1 presents the characteristics of the sample. The average age of the participants was 63 years (*SD* = 11). The sample was predominantly white (84.6%) with an even distribution of sex (female = 52.3%). A majority had been or were smokers (53%), overweight (average BMI = 28.16 kg/m^2^), and prehypertensive (average SBP = 127 mmHg). Participants were taking anti-hypertensive medication (40.1%), oral hypoglycemic medication (4.1%), lipid-lowering medication (12.1%), and insulin (1.6%). A small percentage of the participants had a history of CABG (3.7%) and coronary angioplasty (3.3%). On average, participants slept for 6 hours (= 359.84 min) per night, of which 32.4% were in the supine position. Their sleep was efficient based on the sleep efficiency ratio (82.8%) and mild in the apnea-hypopnea index (13.7). The mean frequency of angina episodes since baseline was 0.48 with 43.6% of participants reporting at least one angina episode (range: 0–7).

**Table 1:** Characteristics of the sample (*N* = 5,804)

Variable	M	SD	*n*	%
Age (year)	63.14	11.22		
Race				
White			4907	84.6
Black			515	8.9
Other			380	6.5
Sex				
Male			2765	47.7
Female			3037	52.3
Smoking				
Never			2708	47.0
Former			2495	43.3
Current			560	9.7
Stress				
Less than usual			1009	17.6
Usual – typical day			3869	67.5
More stressful			850	14.8
BMI (kg/m^2^)	28.16	5.09		
SBP (mmHg)	127.38	19.33		
Diabetes				
Yes			405	7.3
No			5125	92.7
Anti-hypertensive medication				
Yes			2319	40.1
No			3467	59.9
Oral hypoglycemic medication				
Yes			236	4.1
No			5550	95.9
Lipid-lowering medication				
Yes			698	12.1
No			5088	87.9
Insulin				
Yes			91	1.6
No			5695	98.4
Cholesterol (mg/dL)	207.56	38.67		
CABG				
Yes			214	3.7
No			5558	96.3
Coronary angioplasty				
Yes			188	3.3
No			5576	96.7
Sleep duration (minute)	359.84	64.56		
Sleep duration in supine position (percent)	32.40	33.14		
Sleep efficiency	82.77	10.55		
Apnea-hypopnea index	13.72	14.94		
Frequency of angina	0.48	0.64		
None			2842	56.4
1–7			2200	43.6

*BMI *body mass index; *SBP* systolic blood pressure; *CABG* coronary artery bypass grafting

Table 2 shows bivariate associations of angina frequencies with potential correlates. Frequent angina was correlated with older age (*p* <.001), white race (*p* =.004) or other races than black (*p* <.001), former smoking status (*p* =.005), experiencing usual stress (*p* =.036), higher BMI (*p* <.001), diabetes (*p* =.003), use of anti-hypertensive medication (*p* <.001), lipid-lowering medication (*p* =.038), insulin use (*p* =.001), history of CABG (*p* <.001), history of coronary angioplasty (*p* <.001), shorter sleep duration (*p* =.007), longer sleep duration in supine position (*p* <.001), and higher apnea-hypopnea index (*p* =.041).

**Table 2:** Bivariate analyses of frequency of angina using simple Poisson regression

Parameter	B	SE	p*	Exp(B)	95% CI for Exp(B)	
					Lower	Upper
Age (year)	0.016	0.0018	< 0.001	1.016	1.013	1.020
Race (ref = white)						
Black	−0.257	0.0883	0.004	0.774	0.651	0.920
Other	−2.738	0.3022	< 0.001	0.065	0.036	0.117
Female (ref = male)	−0.063	0.0406	0.118	0.939	0.867	1.016
Smoking (ref = never)						
Former	0.120	0.0426	0.005	1.127	1.037	1.225
Current	−0.017	0.0739	0.818	0.983	0.851	1.136
Stress (ref = less than usual)	−0.009	0.0743	0.901	0.991	0.856	1.146
Usual – typical day	0.116	0.0553	0.036	1.123	1.008	1.251
More stressful	0.013	0.0737	0.860	1.013	0.877	1.170
BMI (kg/m^2^)	0.017	0.0039	< 0.001	1.017	1.009	1.025
SBP (mmHg)	−0.001	0.0011	0.252	0.999	0.997	1.001
Diabetes (ref = no)	0.209	0.0709	0.003	1.233	1.073	1.417
Anti-hypertensive medication (ref = no)	0.171	0.0410	< 0.001	1.186	1.095	1.286
Oral hypoglycemic medication (ref = no)	0.153	0.0922	0.097	1.166	0.973	1.396
Lipid-lowering medication (ref = no)	0.121	0.0585	0.038	1.129	1.007	1.266
Insulin (ref = no)	0.414	0.1277	0.001	1.513	1.178	1.944
Cholesterol (mg/dL)	0.000	0.0005	0.567	1.000	0.999	1.001
CABG (ref = no)	0.322	0.0908	< 0.001	1.380	1.155	1.648
Coronary angioplasty (ref = no)	0.514	0.0898	< 0.001	1.672	1.402	1.994
Sleep duration (minute)	−0.001	0.0003	0.007	0.999	0.999	0.999
Sleep duration in supine position (percent)	0.004	0.0006	< 0.001	1.004	1.003	1.005
Sleep efficiency	0.000	0.0019	0.847	1.000	0.996	1.003
Apnea-hypopnea index	0.003	0.0013	0.041	1.003	1.001	1.005

ref = reference category; BMI = body mass index; SBP = systolic blood pressure; CABG = coronary artery bypass grafting; * two-tailed

In a multiple Poisson regression model (see Table 3), frequent angina was correlated with older age (*p* <.001), white race (*p* <.001) or other races compared with black (*p* <.001), higher BMI (*p* <.001), higher SBP (*p* =.002), insulin use (*p* =.045), history of coronary angioplasty (*p* <.001), shorter sleep duration (*p* =.033), longer sleep duration in supine position (*p* <.001), higher sleep efficiency (*p* =.009) and higher apnea-hypopnea index (*p* =.047). In particular, longer sleep duration in the supine position was associated with an increased risk of angina (IRR = 1.003) when controlling for other covariates in the model. Former smoking status, experiencing usual stress, diabetes, use of anti-hypertensive medication or lipid-lowering medication, and history of CABG, which were significant correlates in their bivariate associations, were not found to be significant correlates in the multiple Poisson regression model. Instead, higher SBP and higher sleep efficiency emerged as significant.

**Table 3:** Multiple Poisson regressions on frequency of angina

Parameter	B	SE	p*	Exp(B)	95% CI for Exp(B)	
					Lower	Upper
Intercept	−1.799	0.3282	< 0.001	0.165	0.087	0.315
Age (year)	0.010	0.0023	< 0.001	1.010	1.005	1.014
Race (ref = white)						
Black	−0.329	0.0919	< 0.001	0.719	0.601	0.861
Other	−2.487	0.3037	< 0.001	0.083	0.046	0.151
Female (ref = male)	−0.048	0.0450	0.282	0.953	0.872	1.041
Smoking (ref = never)						
Former	0.047	0.0444	0.293	1.048	0.960	1.143
Current	0.078	0.0756	0.305	1.081	0.932	1.253
Stress (ref = less than usual)	−0.009	0.0743	0.901	0.991	0.856	1.146
Usual	0.019	0.0564	0.737	1.019	0.912	1.138
More stressful	−0.009	0.0743	0.901	0.991	0.856	1.146
BMI (kg/m^2^)	0.024	0.0044	< 0.001	1.024	1.016	1.033
SBP (mmHg)	−0.004	0.0012	0.002	0.996	0.994	0.999
Diabetes (ref = no)	0.032	0.1093	0.773	1.032	0.833	1.279
Anti-hypertensive medication (ref = no)	0.045	0.0465	0.329	1.046	0.955	1.146
Oral hypoglycemic medication (ref = no)	−0.022	0.1259	0.859	0.978	0.764	1.252
Lipid-lowering medication (ref = no)	−0.027	0.0623	0.661	0.973	0.861	1.100
Insulin (ref = no)	0.312	0.1555	0.045	1.367	1.008	1.853
Cholesterol (mg/dL)	0.000	0.0006	0.740	1.000	0.999	1.001
CABG (ref = no)	0.089	0.0997	0.371	1.093	0.899	1.329
Coronary angioplasty (ref = no)	0.349	0.0984	< 0.001	1.417	1.169	1.719
Sleep duration (minute)	−0.001	0.0004	0.033	0.999	0.998	0.999
Sleep duration in supine position (percent)	0.003	0.0006	< 0.001	1.003	1.002	1.005
Sleep efficiency	0.007	0.0026	0.009	1.007	1.002	1.012
Apnea-hypopnea index	−0.003	0.0015	0.047	0.997	0.994	0.999

ref = reference category; BMI = body mass index; SBP = systolic blood pressure; CABG = coronary artery bypass grafting; * two-tailed

In our examination of moderating effects, BMI (*p* =.189) and sex (*p* =.117) did not moderate the association between sleep duration in the supine position and frequency of angina (see Table 4).

**Table 4:** Moderating effect of body mass index and sex on the association between sleep duration in supine position and frequency of angina

Variable	B	SE	p*	Exp(B)	95% CI for Exp(B)	
					Lower	Upper
Sleep duration in supine position (percent) and BMI	0.001	0.0005	0.189	1.001	1.000	1.002
Sleep duration in supine position (percent) and sex	−0.002	0.0012	0.117	0.998	0.996	1.000

BMI = body mass index; Covariates in the models included age, race, sex, smoking, stress, BMI, systolic blood pressure, diabetes, anti-hypertensive medication, oral hypoglycemic medication, lipid-lowering medication, insulin, cholesterol, coronary artery bypass grafting, coronary angioplasty, sleep duration, sleep duration in supine position, sleep efficiency, and apnea-hypopnea index; * two-tailed

## Discussion

### Study findings

This study marks the initial exploration, to the best of our knowledge, of the link between sleeping position and angina episodes in a longitudinal context. Analyzing the large data from a group of 5,804 individuals across the U.S., we discovered that the proportion of time spent in the supine position during sleep is significantly associated with the frequency of angina episodes over a 15-year follow-up period. The finding suggested that a 10% increase in time spent sleeping in the supine position is associated with a 3% increase in the risk of angina, and this association was independent of the conventional CVD risk factors. Notably, this correlation remained consistent regardless of BMI and sex, known risk factors for angina that could potentially influence the effect of sleep position on CVD risk. The average prevalence of angina in our study was less than one episode with a range of 0 to 7, compared to the rates of 7.6% to 21.2% reported in two studies describing angina prevalence among patients with chronic CAD [27, 28]. This lower angina rate in our study aligns with the fact that our research was not restricted to patients with symptomatic or obstructive CAD. Nevertheless, our study shows that 43.6% of participants experienced angina over the 15-year period. This prevalence rate is notably higher than the 3.9% reported for non-institutionalized U.S. adults aged 20 years or older from the National Health and Nutrition Examination Survey (2017 to 2020) [29] as well as 8% in China and 39% in Russia among populations aged 50 or older [30]. Whether this discrepancy arises from differences in the age of the population or the survey period requires further investigation.

### Sleep features and angina

There is mounting evidence indicating that poor sleep quality, abnormal sleep duration, and sleep disorders are linked to a higher risk of CVD [14, 31–35]. This encompasses conditions such as obesity, hypercholesterolemia, hypertension, diabetes, metabolic syndrome, myocardial impaction, and stroke [36]. Several studies have also highlighted the adverse impact of sleep on an elevated risk of CAD [37–39]. However, research investigating the influence of specific sleep characteristics on angina episodes is notably limited. Nevertheless, studies have demonstrated that individuals with very short nightly sleep had more than 3 times more risk of unstable angina in an Iranian population [13]. Additionally, sleep efficiency emerged as a significant predictor of angina, particularly pronounced in hypertensive individuals during an 11-year follow-up study [15]. Interestingly, this relationship was not evident in a cross-sectional analysis [40], suggesting the cumulative and long-term impact of sleep on cardiovascular outcomes. Regarding other sleep characteristics, later bedtime [26], primary central sleep apnea [41], and more than 25% of rapid eye movement (REM) sleep [42] have also been identified as an independent predictor of angina. While no prior studies have explored the correlation between sleep positions and angina, this study suggests that sleep position could be another significant factor influencing angina episodes. The supine position is also known to trigger obstructive events, which have been associated with CVD including hypertension [43], heart failure [44], arrhythmia [45], and cardiovascular events [46]. In this general population, the apnea-hypopnea index was borderline significantly associated with the number of angina episodes. Furthermore, the association between the supine sleep position and angina episodes remained strongly significant after adjusting for the apnea-hypopnea index.

### Sleep positions and previous studies

Previous studies on the impact of habitual sleep positions on health outcomes have mainly focused on sleep positions in infants, during pregnancy, and in positional sleep apnea. For examples, The American Academy of Pediatrics Task Force recommends that infants be placed wholly on their backs to sleep to decrease the risk of sudden infant death syndrome [47]. Sleeping on the left side during pregnancy, especially in the third trimester, is known to improve blood circulation to the fetus and the placenta because it reduces pressure on the inferior vena cava [48, 49]. In positional sleep apnea, the severity of breathing disturbances, such as apneas or hypopneas, is significantly higher when the person is in the supine position compared to other sleeping positions [50, 51]. Additionally, those suffering from gastroesophageal reflux disease are often advised against sleeping on their backs or right side, as these positions can worsen acid reflux [51, 52], while Glaucoma patients may find relief in sleeping in a semi-Fowler’s position as this can help reduce intraocular pressure [51, 53]. Given the significant burden that improper sleep imposes on CVD risk, it is important to note that there is a lack of research exploring the potential influence of sleep positions on cardiovascular function and CVD risk.

### Sleep positions and CV functions

Despite being limited, the few available studies have shown that the right lateral position is associated with a more favorable autonomic profile, characterized by higher parasympathetic and lower sympathetic activity, compared to the supine and left lateral positions. These findings hold across various patient populations, including those with CAD [54], post-CABG surgery [55], normal coronary arteries [19], and heart failure [56, 57], as well as the young adults [19] and the elderly [20]. However, some healthy adult populations show no significant differences in autonomic function across different lying positions [56]. Although the precise mechanisms are yet to be fully comprehended, it’s acknowledged that assuming a right lateral position might enhance venous return and boost cardiac output, potentially leading to reduced sympathetic activities. Additionally, a study has demonstrated that individuals exhibit the lowest plasma norepinephrine concentrations when lying in the right lateral position compared to other positions [57]. Whether the observed association between supine sleep time and angina frequency reflects mechanisms such as altered autonomic tone or hemodynamic changes in the supine position remains an open question that merits future exploration. Recently, the glymphatic system has garnered significant attention due to its crucial role in brain health and function. An animal study has demonstrated that glymphatic transport was most efficient in the lateral position compared with the supine or prone positions [58], suggesting that habitual sleep positions may also play a role in glymphatic function. As glymphatic flow is driven by arterial pulsatility and closely tied to vascular health [59], individuals with higher CVD risk may experience compromised waste clearance during sleep, potentially accelerating the accumulation of neurotoxic metabolites [60]. Future studies should investigate how the combined effects of elevated CVD risk and impaired glymphatic clearance in the supine position may accelerate the development of neurodegenerative diseases. The current dataset provides information only on supine and non-supine positions, lacking details about the proportion of time in either the right or left sleep position. Nonetheless, our study findings underscore the potential importance of identifying recommended sleep positions for cardiovascular health.

### Study strengths and limitations

The strength of the present study includes a large number of patients across a geographically broad range of the U.S., the use of reliable measures of sleep, the adjustment for comprehensive CV risk factors, and the longitudinal follow-up of outcome variable over 15 years of time. Despite these notable advantages, our study should be interpreted in the context of the following potential limitations. First, due to the retrospective nature of the study and the analysis methods employed, the findings suggest an association rather than a causal relationship, and therefore, causal inferences should be avoided. Secondly, the predominantly white and older adult population may limit the generalizability of the findings to more ethnically diverse and younger populations within the U.S. Thirdly, the measurement of sleep position is confined to the proportion of time spent in the supine position, overlooking other sleep positions such as right/left lateral and prone positions. Consequently, there may be unexplored associations in the data. Fourthly, the duration of sleep position was based on just one night. To better characterize habitual sleep positions, it is important to consider data collected over an extended period. Fifthly, the outcome variable focuses on the total number of angina episodes without specifying the types of angina experienced. This lack of specificity could potentially obscure important distinctions in the severity or nature of the condition, impacting the interpretation of the results. Lastly, despite the efforts to account for various potential confounders, there remains a possibility of residual confounding. For instance, the study lacks information on variables such as anxiety and physical activity, which are known to be associated with angina episodes. This missing data could introduce bias and affect the validity of the conclusions drawn from the analysis.

## Conclusion

Our study finding suggests that the proportion of time spent in the supine sleep position during sleep serves as an independent predictor of angina episodes, indicating its potential influence on cardiac health. Previous studies have highlighted the possible advantages of adopting the right lateral position during sleep, demonstrating its ability to reduce cardiac sympathetic nerve activity and fostering an enhanced sympatho-vagal balance, both critical for optimal heart function and regulation. While these findings offer valuable insights, additional studies are imperative to explore the underlying mechanisms that link sleep positions and angina episodes. It’s important to acknowledge that maintaining a single position throughout the night may not be feasible for most individuals. Nonetheless, understanding how specific positions during sleep affect heart function could lead to more personalized recommendations for improving cardiac health. These forthcoming investigations may provide invaluable insights into establishing ideal and suggested sleep positions conducive to promoting cardiovascular health.

## Data Availability

The data from the Sleep Heart Health Study are publicly available through the National Sleep Research Resource (NSRR) and can be accessed at https://sleepdata.org/datasets/shhs, following the data-sharing policies outlined by the NSRR.
